# Epigenetic Control of Cell Potency and Fate Determination during Mammalian Gastrulation

**DOI:** 10.3390/genes14061143

**Published:** 2023-05-25

**Authors:** Adrienne E. Sullivan

**Affiliations:** 1 Quantitative Stem Cell Biology Lab, Francis Crick Institute, London NW1 1AT, UK; adrienne.sullivan@adelaide.edu.au; 2 Adelaide Centre for Epigenetics, School of Biomedicine, Faculty of Health and Medical Sciences, University of Adelaide, Adelaide 5000, Australia; 3 South Australian immunoGENomics Cancer Institute (SAiGENCI), Faculty of Health and Medical Sciences, University of Adelaide, Adelaide 5000, Australia

**Keywords:** embryonic stem cells, epigenetic remodelling, lineage specification, embryonic development, gene regulation

## Abstract

Pluripotent embryonic stem cells have a unique and characteristic epigenetic profile, which is critical for differentiation to all embryonic germ lineages. When stem cells exit the pluripotent state and commit to lineage-specific identities during the process of gastrulation in early embryogenesis, extensive epigenetic remodelling mediates both the switch in cellular programme and the loss of potential to adopt alternative lineage programmes. However, it remains to be understood how the stem cell epigenetic profile encodes pluripotency, or how dynamic epigenetic regulation helps to direct cell fate specification. Recent advances in stem cell culture techniques, cellular reprogramming, and single-cell technologies that can quantitatively profile epigenetic marks have led to significant insights into these questions, which are important for understanding both embryonic development and cell fate engineering. This review provides an overview of key concepts and highlights exciting new advances in the field.

## 1. Introduction

Embryonic stem cells (ESCs) are defined by their remarkable ability to differentiate to all cell types of the embryo proper. In vivo, pluripotent cells of the post-implantation epiblast differentiate to form the three embryonic germ layers (mesoderm, endoderm, and ectoderm) and generate the embryonic body plan during the highly spatiotemporally coordinated process known as gastrulation. The differentiation of epiblast cells is, therefore, precisely controlled to ensure robust exit from the pluripotent state and accurate specification to new identities. The molecular basis for the property of pluripotency, as well as how cells undergo a controlled transition from this state during lineage specification, are still key questions in developmental biology. Answering these questions is important for understanding this critical stage of development, but also, more broadly, how a cell state is defined and manipulated which has significant implications for cell fate engineering technologies. Decades of research have made it clear that the unique epigenetic state of ESCs plays a central role in these processes.

This review provides an overview of the current understanding of key epigenetic features that underly pluripotency, how this epigenetic landscape is remodelled during differentiation, and how remodelling mediates the processes of fate choice and lineage specification. While there are many epigenetic mechanisms which contribute to cell identity, this review will focus specifically on DNA methylation, bivalent and primed chromatin, and nucleosome remodelling. I highlight recent advances in the field and discuss current gaps in understanding and future areas of research. This review complements recent excellent reviews, which provide more comprehensive detail of the specific mechanisms discussed here [1,2,3,4,5].

### 1.1. Embryonic Stem Cells Are a Useful Model to Study Epigenetic Regulation during Gastrulation

Epigenetic regulation of gene expression is critical for enforcing different cellular transcriptomic programs, to the extent that the genome-wide epigenetic profile is a powerful metric for defining cell types [6,7]. In general, for a gene to be efficiently transcribed, both the gene itself and corresponding promoter and regulatory enhancer regions need to be physically accessible (i.e., not densely wrapped in nucleosomes) and permissive to binding oftranscriptional machinery. The interactions between DNA, histone proteins, and transcription factors are all regulated through chemical modifications, which form an epigenetic ‘code’ that both informs and reflects the expression status of genes (Table 1). Studies have shown that ESCs have a uniquely and characteristically high degree of genome-wide chromatin accessibility, but this does not correlate with the identity or diversity of genes that are actively expressed [8]. Rather, the epigenetic landscape of ESCs is required to be permissive of all the potential future lineage choices that are made by ESCs during embryonic development. As part of the cell fate decision process, the pluripotent epigenome undergoes extensive remodelling to mediate: (1) the downregulation of the pluripotency transcriptional program, (2) upregulation of the lineage-specific transcriptional program, and (3) loss of potency for other lineages through remodelling of inactive but permissive chromatin [6,9,10,11].

**Figure 1:**
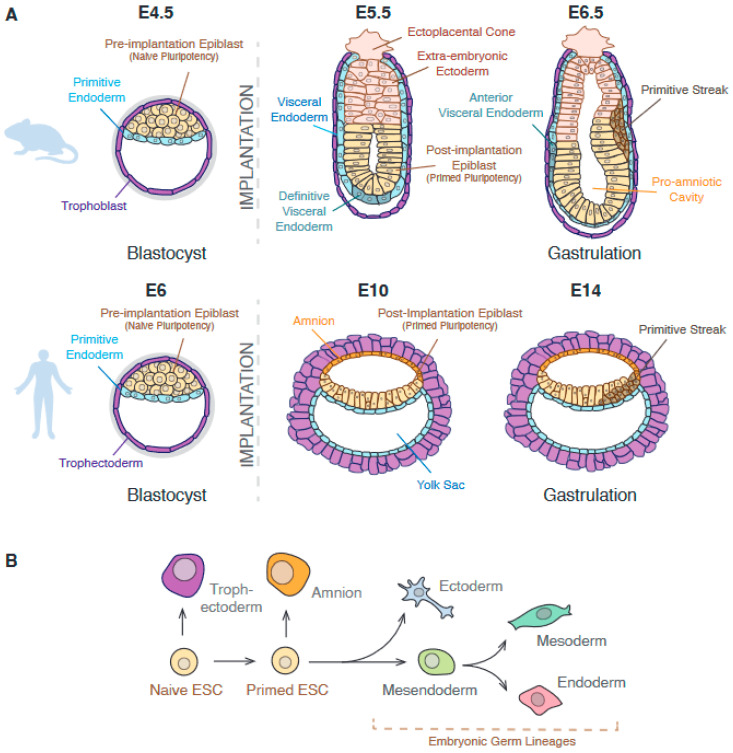
(**A**) Schematics showing the structure of mouse (top) and human (bottom) embryos from pre-implantation to early gastrulation. The primitive streak structure is formed during gastrulation, where epiblast cells differentiate into embryonic germ lineages. (**B**) Schematic showing general differentiation trajectories during early embryonic development, where mouse and human primed ESCs can form the three germ layers: ectoderm, mesoderm, and endoderm. Mesoderm and endoderm share a progenitor state known as mesendoderm. Naïve hESCs can also form trophectoderm-like cells in culture, whereas primed hESCs form extra-embryonic amnion.

Cultured mammalian ESCs are often utilised as a model system to study how pluripotency is encoded and interpreted epigenetically, as well as how dynamic regulation of the epigenetic profile helps direct lineage specification. Both human (h) and mouse (m) cultured ESCs were originally derived from the inner cell mass of pre-implantation blastocysts [12,13], which exist in a state known as ‘naïve’ pluripotency that is now understood to be transcriptionally and epigenetically distinct from post-implantation ‘primed’ pluripotency [8,14,15] (Figure 1A). Cultured mESCs have historically been, and still often are, maintained in a state of naïve pluripotency via culture in media supplemented with LIF (leukemia inhibitory factor) and 2i (GSK3Beta inhibitor and MAPKK inhibitor), or as a heterogeneous, partially naïve population in media with serum and LIF. Primed mESCs, also referred to as ‘epiblast stem cells’ (EpiSCs), can be generated by culturing naïve mESCs in media containing serum alone or with Activin/FGF2. Conversely, it is now understood that cultured hESCs more closely resemble primed pluripotency [16]. Both mESCs and hESC cultures can be induced to form all embryonic germ lineages and some extra-embryonic cell types (Figure 1B) [17], which makes them ideal to interrogate the interplay between epigenetic regulation and developmental fate decisions during gastrulation.

### 1.2. Both Active and Silenced: A State of Gene Regulatory Regions Which Is Critical for Pluripotency

**Figure 2:**
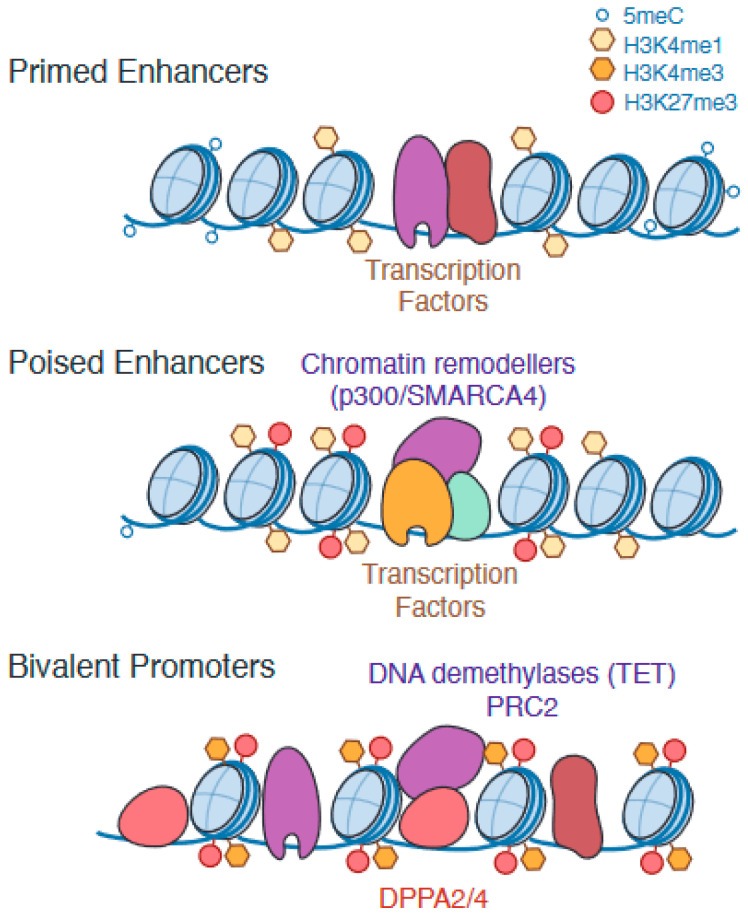
The regulatory regions of various differentiation genes in ESCs are epigenetically marked to ensure robust future expression. Based on these epigenetic modifications, some enhancer regions are classed as ‘primed’ or ‘poised’, and promoters which are enriched for both activating and repressing histone modifications are ‘bivalent’.

A key characteristic of the ESC epigenome is the ‘bivalent’ promoter—a promoter that is enriched for both activating and silencing epigenetic marks ([18,19], reviewed in [1]) (Figure 2). These regions are characterised by a cooccurrence of H3K4me3 (marker of active promoters and established by KMT2B/MLL2 as part of the COMPASS-like complex) and H3K27me3 (marker of silencing by Polycomb repressive complex PRC2) and are associated with minimal levels of expression in pluripotent cells (Table 1). Bivalent promoters have been observed for various differentiation and lineage-associated genes in pluripotent stem cells, and, accordingly, the bivalent state can resolve to active or silenced as a cell undergoes lineage specification [18,19]. Perturbing bivalency to study the impact on gene regulation has traditionally been challenging, as the complexes which establish histone marks on bivalent promoters regulate various other sites on the genome as well. However, knockout (KO) of Kmt2b to deplete H3K4me3 in mouse embryos results in post-implantation embryonic lethality [20], and *Kmt2b* KO mESCs demonstrate impaired activation of bivalent genes during embryoid body formation [21,22]. Bivalency is, therefore, thought to play a critical role in the proper regulation of key developmental genes.

Although the molecular mechanisms controlling the establishment, maintenance, and resolution of bivalent regions are still not well understood, one such mechanism was identified in a study that found that pluripotency factors Dppa2/4 are required for bivalency in a subset of promoters in mESCs [23]. Knockout of Dppa2/4 resulted in loss of or reduction in both active and inactive histone marks followed by a gain in DNA methylation at bivalent promoters, and associated genes were subsequently unable to be upregulated during mESC differentiation. When Dppa2/4 levels were transiently knocked down and then recovered, bivalency was restored. This study established the concept of ‘epigenetic priming factors’, which actively and directly maintain features such as promoter bivalency for the sake of future gene expression [24].

Similar to bivalent promoters, enhancer regions in ESCs can also exist in a state that is neither active nor fully silenced [25] (Figure 2). ‘Primed’ enhancers are enriched for active enhancer mark H3K4me1 and have low nucleosome density but lack both the active mark H3K27ac and the silencing mark H3K27me3. A similar but distinct class of enhancers termed ‘poised’ is characterised by chromatin accessibility and the enrichment of both H3K4me1 and H3K27me3 but not H3K27ac [26]. Interestingly, H3K27me3 is not solely responsible for keeping poised enhancers inactive, as a study which inhibited PRC2 activity in mESCs observed a decrease in H3K27me3 at poised enhancers but not a corresponding increase in H3K27ac [27].

Both poised and primed enhancers are proximal to genes that are largely inactive in pluripotency but become expressed during the processes of gastrulation and early embryogenesis [25,26]. Accordingly, these enhancers can become active or silenced as cells differentiate, and it has been shown both in vitro and in vivo that the activity of these enhancers is important for proper specification to embryonic lineages [25,26,27,28]. In the pluripotent state, poised and primed enhancers are hypomethylated and frequently occupied by pluripotency factors, such as POU5F1 (OCT4) and SOX2 [25,29]. One study observed that when the binding sites of pluripotency factors Esrrb or Sox2 were removed from candidate inactive enhancers by CRISPR-mediated genome editing in mESCs, both the activity of the edited enhancers and upregulation of the associated genes were impaired in the resulting mESC-derived differentiated cell types, demonstrating that the binding of pluripotency factors is important for the future activity of these enhancers [29]. As with promoter bivalency, keeping developmentally important enhancers accessible and permissive to activation appears to be a critical mechanism underlying the potency of stem cells.

A major question that remains to be fully understood, however, is why particular enhancers and promoters must exist in these distinct epigenetic states to ensure their timely and robust induction during embryogenesis. As many of the associated genes are induced early in gastrulation, a plausible explanation is that poising and bivalency allow for rapid upregulation of gene expression in response to differentiation cues. This hypothesis is supported by the observation that bivalent promoters are frequently occupied by paused polymerases [30], which, in other contexts, mediate the rapid and synchronised onset of transcription [31]. However, a recent study of differentiation of naïve mESCs to EpiLCs observed that genes with bivalent promoters (H3K4me3 and H3K27me3) are not upregulated faster than genes with repressed promoters (H3K27me3 alone) [32]. An alternative hypothesis is that bivalency protects a subset of CpG-rich promoters from silencing through de novo DNA methylation while still maintaining transcriptional repression; methylation of H3K4 is known to inhibit the activity of DNMT3 [33,34], and bivalent promoters gained DNA methylation when H3K4me3 was depleted in mESCs through knockout of Kmt2B [35]. It is possible that a similar mechanism operates for CpG-rich enhancers. Additionally, bivalent or poised chromatin could be required to recruit specific transcription factors during differentiation [36], help control absolute levels of gene expression, or potentially affect the rate and efficiency of decommissioning regulatory regions during lineage specification.

### 1.3. Epigenetic Remodelling of Enhancers Is Required for Successful Pluripotency Exit and Robust Cell Fate Specification

**Figure 3:**
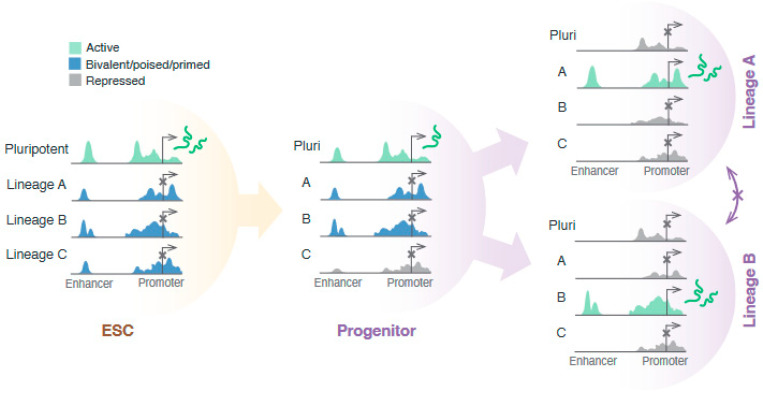
Pluripotent cells express genes associated with the pluripotent state and keep other genes associated with potential lineages inactive but accessible. An example theoretical lineage trajectory is shown. Cells that have been induced to differentiate start downregulating pluripotency genes and upregulating differentiation genes, as well as silencing regulatory regions associated with other lineages. Loss of potency is sequential: progenitors maintain potency for multiple fates while losing potency for others and later undergo further lineage specification.

To effectively transition to a differentiated identity, stem cells must decommission the pluripotency transcriptional and epigenetic programme concomitant with, or in some cases prior to, establishing the lineage-specific programme (Figure 3). A particularly important part of this transition is the switch in cellular enhancer profile, as various studies have identified that the activity and regulation of enhancers, rather than promoters, have a more significant role in embryonic lineage determination [6,9]. This is consistent with observations that enhancers are the most epigenetically dynamic regions in differentiating pluripotent cells [6,10,11]. Several other studies have linked the window of potency for a particular identity with the priming or silencing of the associated enhancer set, which demonstrates that the cellular enhancer profile is predictive of lineage competency [37,38,39,40].

**Figure 4:**
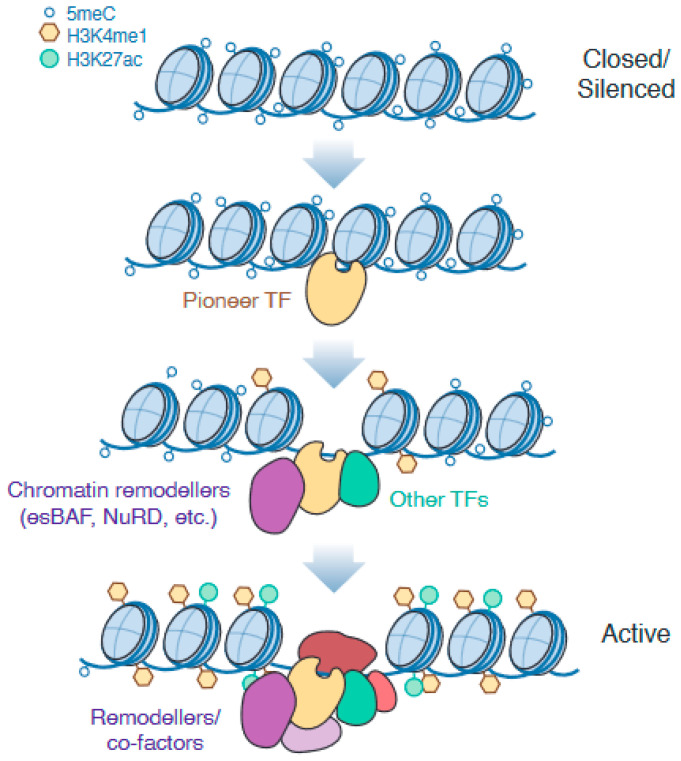
Lineage-specific enhancer regions are established during differentiation through epigenetic remodelling. Pioneer transcription factors interact with silenced chromatin and recruit chromatin remodelling complexes to displace nucleosomes and open the chromatin.

The de novo establishment of accessible enhancers is known to be instigated by the activity of pioneer transcription factors (reviewed in [2]), which are capable of interacting with silenced and inaccessible chromatin to initiate the remodelling and recruitment of further transcriptional machinery [41] (Figure 4). Various key transcription factors that drive enhancer remodelling specific to each germ lineage have been identified [2,10,42], although the molecular basis for pioneer activity is an active area of research. Just as not all transcription factors have pioneer activity, not all genomic sites are accessed when silenced. Studies have shown that the genome-wide binding profile of pioneer factors still varies considerably based on the cellular context and presence of co-binding transcription factors, indicating that pioneer factors also rely on direct or indirect cooperativity of binding for some sites [43]. This means that the role of pioneer factors can evolve as the cell differentiates and the complement of co-binding factors changes, as has been observed for SOX2 [44,45]. Some pioneer factors are also known as ‘master regulators’, such that their expression is both necessary and sufficient to remodel the cellular identity towards a particular fate—these factors are, therefore, utilised in cellular reprogramming techniques [2]. As well as upregulating lineage-specific targets, these regulators can also directly inhibit the transcription of genes associated with alternative lineages, although the mechanisms that determine repressive or activating activity remain unclear. For example, Eomes and T/Brachyury, which are master regulators of meso/endodermal differentiation, also directly repressed neuroectodermal genes in differentiating mESCs [46].

Nucleosome remodelling activity is required to both establish and maintain enhancer accessibility. In particular, ESCs express a specific SWI/SNF nucleosome remodelling complex known as esBAF, which is essential for maintaining pluripotency [47]. The main catalytic ATPase subunit of esBAF is SMARCA4/BRG1, found at both active and poised enhancers [26]. Strikingly, the inducible degradation or inhibition of SMARCA4 causes enhancers to rapidly become inactive and inaccessible [48,49]. This effect was seen regardless of the cell cycle phase and was readily reversible in mESCs [48]. SMARCA4 is also known to be directly recruited by master pluripotency factors such as POU5F1 [50] and differentiation pioneer factors such as GATA3 [51], demonstrating its importance in both maintaining pluripotency and establishing differentiated identities.

In comparison to enhancer and promoter activation, less is known about the molecular mechanisms involved in enhancer decommissioning. Several perturbation studies have shown that maintaining enhancer accessibility is an active process, and when enhancer-bound transcription factors and associated activation complexes are removed through depletion [49], inhibition [48], or mutation of binding sites [29], enhancers quickly become silenced. It has also been proposed that a balance exists between the activity of co-bound activating transcription factors and chromatin silencing machinery, such as histone demethylase KDM1A (LSD1), which targets the active/primed enhancer mark H3K4me1, or members of the nucleosome remodelling de-acetylase (NuRD) complex [52,53]. This balance of activity would then be disrupted when transcription factors are downregulated or displaced, resulting in silencing. Silencing machinery is known to be important for decommissioning pluripotent enhancers, as Kdm1A KO mESCs that were differentiated by depleting Pou5f1 [52] upregulated differentiation genes but failed to completely downregulate various pluripotency markers or adopt a fully differentiated morphology. Further work is needed to fully test this hypothesis, as well as determine whether and how enhancer subsets can be selectively targeted for silencing. Given that ESCs have a large complement of enhancers that are then selectively remodelled during lineage specification, it is likely that global perturbation approaches will not be as informative as locus-specific methods in answering these questions.

### 1.4. Redistribution of Repressive Histone Marks Is Important for Fidelity of Lineage Choice

Remodelling repressive epigenetic marks to mediate the switch in cellular programmes is also a critical element of ESC differentiation, as various studies have reported gastrulation defects in mouse embryos when H3K27me3 is globally depleted as a result of PRC2 inhibition [54,55,56]. In particular, H3K27me3 is known to enforce cell states by preventing ectopic expression of silenced genes—when H2K27me3 was depleted in ESCs through knockout of core subunit(s) of the PRC2 complex such as EZH2, proliferation was reduced, and the sporadic and spontaneous upregulation of differentiation genes was observed in primed but not naïve ESCs [57,58]. Interestingly, directed differentiation of EZH2 KO hESCs successfully downregulated pluripotency genes and upregulated differentiation genes, indicating that H3K27me3 is not essential for repression of the pluripotency network [57]. However, differentiation genes were more highly upregulated than in WT cells, and some ectopic expression of genes associated with alternative lineages was observed. Similar observations have been made for PRC2-inhibited mESCs, which also successfully repressed pluripotency genes during differentiation but displayed increased upregulation of lineage markers and ectopic marker expression [27,59]. Interestingly, a more recent study utilised an inducible degradation system to rapidly and acutely deplete PRC2 subunits and observed de-repression of both alternative lineage genes and pluripotency-expressed genes in mESC-derived neural progenitors [60]. These studies demonstrate a clear role for PRC2 and H3K27me3 in controlling the robustness of cell fate induction and insulation from other lineage programs, but it is less clear how important H3K27me3 is for repression of the pluripotency programme in differentiating cells. The reported differences between constitutive knockout and inducible degradation systems may be due to compensation effects, which warrants further investigation.

### 1.5. DNA Methylation Plays a Key but Nuanced Role in Silencing of Gene Regulatory Regions

Methylation of 5-cytosine of CpG DNA moieties (5meC) is well established as an important mechanism of transcriptional silencing used to suppress the expression of retrotransposons, mediate X-chromosome inactivation, and control the activity of gene regulatory regions during development through dynamic remodelling (reviewed in [3]). The cellular DNA methylation profile undergoes several rounds of extensive remodelling during embryonic development; in both mice and humans, DNA is globally demethylated after fertilisation but becomes remethylated in the post-implantation epiblast [61,62], correlating with the increased expression of DNA methyltransferase proteins DNMT3A and DNMT3B, which are responsible for de novo DNA methylation. As epiblast cells undergo fate specification during gastrulation, DNA methylation is deposited or erased accordingly at lineage-specific loci [6,10,11]. However, several recent studies have questioned the strict importance of DNA methylation in gatekeeping gene expression during embryonic development.

When demethylation is ablated in mouse embryos via triple knockout (TKO) of all ten-eleven translocation (TET) dioxygenases (TET1-3), various critical pro-differentiation genes are misregulated, including factors which affect essential Wnt and Nodal signalling pathway activity. As a result, significant defects and early embryonic lethality occur in gastrulating embryos [63,64]. To examine the cell-intrinsic effects of demethylation during gastrulation that may be masked by the gross defects caused by misregulation of signalling, Cheng et al. created chimeric embryos from Tet-TKO mESCs injected into wildtype blastocysts in order to allow Tet-TKO cells to develop within a primarily WT embryonic signalling environment [65]. Some signalling-based developmental defects were still observed when a wholly Tet-TKO epiblast developed with WT extra-embryonic tissues, but this phenotype was almost entirely rescued when both WT and Tet-TKO cells contributed to the epiblast population. Rescued TKO cells in chimeric epiblasts were able to contribute to all lineages except for notochord, but still displayed autonomous gene misregulation that primarily manifested as altered quantitative levels of expression. Interestingly, this indicates that DNA methylation may generally function to modulate expression levels rather than outright block gene induction during gastrulation.

The interplay between DNA methylation and transcription factor binding, where binding of transcription factors prevents de novo methylation but DNA methylation can inhibit the binding of various transcription factors, presents a challenge in determining cause and effect for changes in DNA methylation and enhancer activity. This relationship has recently been interrogated at the molecular level using novel single-molecule foot-printing techniques in mESCs [66]. Contrary to measurements made using bulk cell populations, foot-printing analysis of enhancer regions did not find a universal correlation between 5meC levels and chromatin inaccessibility and, in fact, observed that the activity of most enhancers in mESCs is not affected by methylation levels. Only a subset of enhancers had the expected inverse relationship of DNA methylation and accessibility, which also held true as mESCs were differentiated to neural cell types and methylation of these sites increased as accessibility decreased. Manipulating global DNA methylation through the knockout of all enzymes that either write and maintain 5mC (DMNT1/3A/3B TKO) or erase it (TET TKO) caused both the accessibility and activity of these enhancers to decrease or increase, respectively [66,67], indicating that this relationship is causative and likely mediated by altered binding of methylation-sensitive transcription factors.

Experiments in vitro and in vivo have shown that methylation sensitivity differs greatly between different transcription factors, but also that a transcription factor can display selective or partial sensitivity in context, for example, if only some variants of recognised DNA motifs contain a CpG, or depending on the position of CpGs relative to the motif [66,67,68]. Other factors which contain Methyl-CpG binding domains (MBDs) bind methylated DNA in a sequence non-specific manner, although a recent knockout study has shown that recruitment of these proteins is largely dispensable for transcriptional repression in mESCs [67].

These studies describe a surprisingly nuanced role for DNA methylation in the regulation of developmental enhancers. Depending on the specific enhancer and transcription proteins involved, DNA methylation ranges from seemingly dispensable, to important for dampening maximal levels of gene expression, to silencing enhancer activity outright. This creates an additional level of selective regulation that can aid in the fine-tuning control of enhancer activity and, hence, gene expression programs.

### 1.6. How Can Epigenetics Influence Differentiation Trajectories of Multipotent Cells and Vice Versa?

As previously discussed, remodelling of the cellular epigenome is required for successful lineage specification and loss of potency in differentiating ESCs. However, it remains to be fully understood how and to what extent the cell epigenetic profile can influence fate choice. A common concept in the biology of pluri- or multipotent progenitor cells is that of an inherent ‘default’ path of differentiation, which is the trajectory undertaken when the progenitor state is destabilised but no inductive cues are present to direct fate choice. For primed human and mouse ESCs, the default pathway when cells are removed from pluripotency maintenance media (i.e., FGF and TGFB/NODAL signalling), or when signalling is inhibited, is neuroectoderm [69,70]. Unlike primed hESCs, naïve hESCs—either from blastocyst explants or converted primed cultures—differentiate to extra-embryonic trophoblast-like cells in the absence of signalling, which indicates a default pathway to trophectoderm [17]. This leads to the following question: is the default pathway of differentiation dictated by epigenetic encoding?

There is evidence that mouse epiblast cells display epigenetic priming towards the default lineage of the ectoderm. In single-cell multiomics (mRNA, DNA methylation, chromatin accessibility) data from mouse embryos, endoderm- and mesoderm-associated enhancers increased in accessibility and decreased DNA methylation during lineage specification; conversely, ectoderm-associated enhancers were already accessible prior to gastrulation and underwent silencing in meso/endodermal lineages [6]. Additionally, the majority of poised enhancers in mESCs are active (i.e., gain H3K27ac) in neural progenitors and forebrain in comparison to limb or liver tissues, suggesting lineage bias [27]. For human primed ESCs the evidence is less definitive, with poised and bivalent regulatory regions showing no bias for ectoderm-specific activity in the directed differentiation of cultures [10]. Although further research is required, it is also possible that these differences in epigenetic priming are species-specific.

Based on the idea that ESCs are epigenetically primed towards a specific lineage, it is easy to speculate that inhibiting repressive epigenetic marks will result in differentiation to the default pathway. Although inhibition of PRC2 in naïve hESCs induced differentiation to trophectoderm [14,15], the inhibition of PRC2 activity in both human and mouse primed ESCs caused spontaneous differentiation to endoderm and mesoderm rather than the ectoderm [57,58]. This is primarily due to upregulation of the growth factor BMP4 [58], which drives meso/endoderm differentiation and inhibits neuroectoderm formation at this stage of development [71,72]. These studies demonstrate that the default differentiation pathway in the absence of inductive signalling can be distinct from differentiation due to the atypical condition of global epigenetic de-repression.

Recent studies have shown that the activity of specific epigenetic remodelling factors can exert a cell fate bias in ESCs—for example, the de novo DNA methylase Dnmt3B, but not Dnmt3A, promoted meso/endoderm differentiation through repression of the neuroectodermal gene regulatory regions in primed mESCs [73]. Studies which have depleted alternative subunits of PRC2 also indicate that, while largely redundant in pluripotent mESCs, variant PRC2 complexes have different and fate-biased targets during differentiation [60,74]. Depletion of Jarid2 (PRC2.2) during differentiation to neuroectoderm resulted in de-repression of pluripotency-expressed genes, whereas depletion of Mtf2 (PRC2.1) de-repressed genes that were not expressed in WT mESCs or neural progenitors but are likely active in other lineages [60]. This is consistent with reports that Mtf2 KO mESCs showed increased differentiation to all lineages in embryoid bodies, whereas Jarid2 KO mESCs appear to delay differentiation and down-regulation of pluripotency markers [74]. This indicates that chromatin remodelling machinery is not always neutral to cell lineage and, hence, that regulation of different factors and subunits could bias cell fate decisions.

While the differentiation trajectory taken by a cell can be influenced by its epigenetic profile, the reverse also appears to be true—that the developmental history of a cell can be reflected epigenetically in ways that are not necessarily evident at the transcriptomic level. This concept is the basis for the use of epigenetic profiles to identify the cell type of origin for cancers [75,76] and mesenchymal stem cells [77], and is also observed in induced pluripotency stem cell (iPSC) lines [78]. Epigenetic traces of decommissioned developmental enhancers have also been observed in adult mouse cells as regions of hypomethylated DNA [79]. Remarkably, prolonged PRC2 inhibition successfully reactivated these enhancers and partially reversed the developmental trajectory of the cells, although the purpose of this epigenetic memory in vivo is still unclear.

Additionally, a recent study by Wong et al. observed that the time, as delineated by number of division cycles, spent in a progenitor state affected the cellular epigenetic profile and relative potency for further differentiation [80]. This study differentiated and maintained hESCs in an endodermal progenitor state for a variable amount of time before inducing differentiation to pancreatic endoderm. The authors found that cultures which spent longer as primed progenitors more efficiently decommissioned pluripotent enhancers and increased accessibility of enhancers associated with mature pancreatic fates in preparation for activation, which later correlated with more efficient and robust differentiation to the pancreatic endoderm. The expansion and maintenance of progenitor populations in vivo are, hence, not only important as a resource for building tissue but also for the establishment of an epigenetic landscape primed for future differentiation [80]. In the future, experiments combining methods to track cell lineage and/or expression of key genes with epigenetic profiling techniques such as single cell ATAC-Seq may find further evidence that differentiation trajectory affects the cellular epigenetic profile and thus future cell behaviour. It may also be that trajectory ‘pause points’ such as progenitor expansion are used to correct this variability and ensure a more homogenous epigenetic state in progenitors that progress to mature fates.

## 2. Conclusions and Discussion

Studying the molecular basis for pluripotency loss and lineage specification is particularly challenging because these processes are intrinsically linked, such that perturbation of the pluripotency programme can induce spontaneous differentiation, whereas upregulation of differentiation factors drives the loss of pluripotency. Despite this inherent difficulty, great advances have been made in determining the mechanisms and drivers underlying these processes. It is also clear that coordinating a controlled switch in cellular programmes involves a complex interplay between different systems, and there is still much to understand about these processes and how they relate to cellular outcomes in vitro and in vivo.

It must be noted that many historic findings in the field were made before naïve and primed pluripotency were fully appreciated as distinct states, and default culture conditions for mESCs and hESCs maintain states of naïve and primed pluripotency, respectively. More recently, methods to induce hESCs to a naïve state [81], as well as comparative naïve/primed mESC analysis, are being used to fully clarify species- and cell-state-specific differences. It also remains to be determined whether the epigenetics of human and mouse ESCs reflect the relative timing of lineage formation in vivo; e.g., the formation of amnion from the epiblast occurs prior to gastrulation in primates [82,83] but coincides with gastrulation in mice [84] (Figure 1A).

While directed differentiation protocols to different lineages are useful to study lineage-specific remodelling, advancing technologies have made it possible to study epigenetic and transcriptomic changes at the single-cell level, both in the embryo [6,65] and in tractable, in vivo-relevant models of embryonic patterning and differentiation such as gastruloids and embryoids [85]. Characterising cellular transcriptomic and epigenomic trajectories in the context of a patterning population is critical for understanding how the sequence and timing of epigenetic changes in individual cells or sub-populations helps mediate proper spatiotemporal embryonic development. To this end, novel single-cell sequencing techniques that allow for quantitative multiomics characterization, such as mRNA and histone modifications/protein binding (CUT&Tag) [86], mRNA and chromatin accessibility (ATAC) [87,88], or mRNA, accessibility, and DNA methylation [6,89], are already proving to be particularly powerful. Their continued improvement in both sensitivity and throughput efficiency [90] will surely benefit future work in the field.

The scientific toolbox for observing and perturbing molecular events has also grown considerably in recent years and is providing significant insight into mechanisms of epigenetic regulation. Inducible systems, which allow for rapid, selective control over protein levels, are particularly useful for studying factors which are required for ESC maintenance or have changing roles during lineage specification [49,60]. The ability to interrogate the function and regulation of individual loci through Cas9- or TALE-based epigenetic manipulations [91], or locus-specific proteomics approaches [92,93], is also highly valuable for correlating changes in epigenetics and transcription factor binding with target gene transcription. Given that enhancers exhibit a range of sensitivity to epigenetic modifications and are regulated by various remodelling complexes and transcription factors, having locus-specific data will help clarify regulatory relationships within the context of a particular site.

Additionally, recently developed techniques with single-molecule resolution are providing significant insight into the nature of molecular binding events. Single-molecule foot-printing, which utilises a recombinant DNA methyltransferase to modify physically accessible GpC moieties but not endogenously regulated CpG moieties, has been used to infer properties of transcription factor interactions with DNA, such as order and cooperativity of binding [94] and sensitivity to methylation [66]. These kinds of quantitative molecular data are critical for understanding transcription factor behaviour in vivo, and it will be important to observe how these properties may change as cells differentiate.

How epigenetic regulation encodes cellular identity and potency, and how the controlled and sequential remodelling of the epigenetic profile mediates lineage specification during embryonic development, is a complex subject which involves many layers of inter-regulation. Ultimately, better understanding will come from integrating epigenomic and transcriptomic data together with observations of cellular behaviour and fate transitions, which technological and analytical advances are making increasingly possible.

## Figures and Tables

**Table 1:** A summary of epigenetic marks discussed in this paper, their location, and associated role in regulating gene expression. HATs: Histone Acetyl Transferases, HDACs: Histone deacetylases, SIRTs: sirtuins, PRC2: Polycomb Repressive Complex 2, COMPASS: Complex Proteins Associated with Set1.

Epigenetic Modification	Writers	Erasers	Location	Role
DNA methylation (5meC)	DNMT3A, DNMT3B (maintenance: DNMT1)	TET1, TET2, TET3	CpG	Repression
H3K27ac	HATs, e.g., CBP/p300	HDACs, SIRTs	Active promoters and enhancers	Activation
H3K27me3	PRC2.1, PRC2.2	KDM6A, KDM6B	Repressed and bivalent promoters, poised enhancers	Repression
H3K4me1	COMPASS-like (KMT2C, KMT2D)	KDM1A, KDM1B	Active and poised/primed enhancers	Priming, activating
H3K4me3	COMPASS-like (KMT2A, KMT2B)	KDM5A, KDM5B, KDM5C, KDM5D, KDM2B	Mostly promoters (active and bivalent)	Priming, activating

## Data Availability

No new data was created in this study.
